# How Does the Change of Information Source Affect Residents’ Risk Attitudes?

**DOI:** 10.3389/fpsyg.2022.918427

**Published:** 2022-06-17

**Authors:** Shihu Zhang, Guangcai Zhang, Jinpei Li, Haiying Gu

**Affiliations:** ^1^Business School, Zhengzhou University, Zhengzhou, China; ^2^School of Politics and Public Administration, Zhengzhou University, Zhengzhou, China; ^3^Institute of Applied Economics, Shanghai Academy of Social Sciences, Shanghai, China; ^4^School of Economics and Management, Beijing University of Chemical Technology, Beijing, China; ^5^Antai College of Economics and Management, Shanghai Jiao Tong University, Shanghai, China

**Keywords:** internet use, risk attitudes, uncertainty, information acquisition, decision making

## Abstract

Using data from the China Family Panel Studies (CFPS), this paper investigates the effects of Internet use on residents’ risk attitudes. Both Generalized Ordered Logit Model and Logit model are used to identify the effects of Internet use. The results reveal an association between Internet use and increases in both subjective and objective risk preferences that remains even after we adjust for possible endogeneity. The heterogeneity analysis also reveals that these impacts are different among groups with different reasons for Internet use and different personal characteristics. Our study expands the research on the effects of Internet on people’s concepts from the micro perspective and suggests that while promoting the application of information technology we should also pay attention to the individual characteristics of residents so that we can better share the “digital dividend” brought by the popularization of information technology in the whole society.

## Introduction

Individuals always make decisions under uncertain conditions. Uncertainty brings risks. Individuals’ perceptions and attitudes toward risks are important factors that affect their decision-making behaviors. Risk attitudes shape a broad range of residents’ decisions such as savings, consumption, investment, labor supply, insurance, health services purchase, and many other behaviors (Banks et al., 2019). Although economic theory assumes that individuals’ risk attitudes do not change with time, many studies have found evidence for within-individual variation in risk attitudes over time and show that changes in living and economic circumstances can also affect risk attitudes (Jung and Treibich, 2015; Cho et al., 2018).

Studying the determinants of risk attitudes is conducive to the understanding of individuals’ decision-making behaviors. Theoretically, risk attitudes are directly affected by the quantity and quality of information that individuals can acquire (Tausch and Zumbuehl, 2018). The acquisition of information depends on various information sources, such as the Internet, Television, Newspapers, Magazines, and Social networks. With the continuous improvement of the communication infrastructure and the popularization of intelligent devices, China has become the world’s largest Internet user (Wang and Li, 2012). There are 904 million ‘‘netizens’’ in China as of March 2020.^1^ The country’s Internet economy reached 4.4 trillion US dollars in 2019 and the Internet-fueled GDP growth accounted for 67.9% of China’s total GDP increase in 2019. The Internet has fundamentally changed Chinese daily life. Also, residents’ risk attitudes are significantly influenced by the rapid growth of Internet use in China. Because the popularization of Internet use has expanded the information acquisition sources for residents, increased the density of information, and improved the quality of information, the information acquisition revolution has not only changed various aspects of residents’ life and work, but also had a profound impact on residents’ concepts.

Yet despite the growing importance of Internet use in our daily lives, little attention has been paid to analyzing its influence on residents’ concepts. To the best of our knowledge, the impact of Internet use on the risk attitudes of residents remains understudied. As one of the most important factors affecting residents’ decision-making behaviors, risk attitudes not only play a role at the micro level, but also are closely linked with the innovation ability of the whole society at the macro level. Improving the risk preference of the whole society is conductive to stimulating the innovation vitality of our society. Therefore, exploring the influencing factors of residents’ risk attitudes is of great significance to China’s development strategy of building an innovative country in the future. The aim of this study is to assess the impact of Internet use on residents’ risk attitudes in China. We extend the literature in four ways. First, we provide the first study investigating the influence of Internet use on residents’ risk attitudes. Although a number of studies have examined the factors that affect residents’ risk attitudes, most of them focused on the relationship between fixed individual characteristics and risk attitudes, ignoring the impact of Internet use. Given that China has become the world’s largest Internet user and the information acquisition source of many residents has changed significantly. It is important to analyze the significant impact of Internet use. Second, we employ two different methods: the subjective and objective methods to measure residents’ risk attitudes, which produce a more comprehensive view of the connection between Internet use and risk attitudes. Third, by analyzing not only the specific activities but also the reasons for Internet use and the personal characteristics of Internet users, we provide a valuable perspective to understand the heterogenous impacts of Internet use on residents’ risk attitudes. Fourth, several methods are used to examine the issue of endogeneity in our models, which provide a compelling conclusion that Internet use has an important impact on residents’ risk attitudes.

The rest of this paper is organized as follows. Section “Literature Review” reviews the relevant literature. Section “Data and Methods” describes the data, variables, and methods. Section “Empirical Results” shows the empirical results. Section “Heterogeneity Analysis” provides the heterogeneity analysis. Section “Robustness Analysis” is the robustness analysis. Section “Conclusion and Discussion” concludes.

## Literature Review

### One Important Open Question Is How to Measure Risk Attitudes

Previous studies measure risk attitudes using survey questions, and construct risk attitudes variables by using the respondents’ answers to relevant questions (Gary and Angelino, 2012; Banks et al., 2019), which subjectively evaluates residents’ risk attitudes. The residents’ risk attitudes derived from this method has been proved to have a good ability to explain and predict residents’ risk behaviors (Guiso and Paiella, 2008). But a second question is whether survey questions are really a good method for measuring risk attitudes. Because for respondent survey questions are not incentive compatible, some scholars are skeptical about whether self-reported personal attitudes and traits are behaviorally meaningful (Dohmen et al., 2011). Various factors, including self-serving biases, inattention, and strategic motives could cause respondents to distort their reported risk attitudes (Camerer and Hogarth, 1999). So, given all these limitations, some other experimental studies, which measure risk attitudes with risk-taking behaviors, such as financial investment. These studies also construct residents’ risk attitudes variables through questionnaire data, but they construct risk attitudes variables based on the survey data of residents’ participation in financial markets or holding of risk assets (Cocco, 2005; Dohmen et al., 2011). This method evaluates the objective risk attitudes of residents from the perspective of observing objective behavior. This paper applies the two methods to measure residents’ subjective and objective risk attitudes.

Some studies have found that wealth (Haliassos and Bertaut, 1995), social capital (Wossen et al., 2015), life event shocks, and job or health changes (Sahm, 2012; Banks et al., 2019) all significantly affected residents’ risk attitudes. According to other studies focusing on the relationship between risk attitudes and individual characteristics, residents’ risk attitudes are also significantly related to their individual characteristics, such as age (Gollier, 2002), gender (Dohmen et al., 2011), personality (Bucciol and Zarri, 2017), cognitive ability (Bonsang and Dohmen, 2015), education (Kapteyn and Teppa, 2011; Outreville, 2013), marital status (Arrondel and Lefebvre, 2001), health status (Hammitt et al., 2009), language (Chen, 2013), etc.

Besides, the popularization of Internet use in China has enhanced the ability of residents to acquire information. Their production and operating information and technical knowledge. The information can reduce various uncertainties in their decision-making processes, and thus alleviating their conservative risk aversion attitudes (Ghadim et al., 2005). Yet despite the growing importance of Internet use, in most of the existing studies, there is no direct analysis of the relationship between Internet use and risk attitudes.

Residents’ risk attitudes are directly affected by the information they get (Wijayaratna and Dixit, 2016) and their “information status.” As a new information technology expanding in China, the Internet obviously plays an increasingly important role in affecting residents’ risk attitudes. The different amount of information acquired may lead to people’s different cognitions of specific things. For example, in the outbreak of novel coronavirus pneumonia (COVID-19) at the end of 2019, we can observe that the differences in the knowledge of COVID-19 lead to people’s different risk attitudes to the epidemic (Honarvar et al., 2020), which caused people’s different protective measures to the virus (choose to wear a mask or not; comply with the segregation policy or not). Some other literature also found that the different information set constraints people face when making decisions reflect people’s different “information status”: people usually prefer risk when facing small probability events, but avoid risk when facing high probability events (Fenghua, 2013). The population and application of information technology represented by the Internet have significantly changed the source of residents’ information acquisition and improved their ability of information acquisition. Internet use has impacted residents’ risk attitudes by innovating the information acquisition mode.

Given the importance of Internet use to residents’ risk attitudes, it is essential to analyze and identify its impact. This paper attempts to make some contributions in the following aspects. First, we use microdata to measure the subjective and objective risk attitudes of residents. Through empirical analysis, we verify the influence of Internet use on residents’ subjective and objective risk attitudes. We expand the research on the influencing factors of residents’ risk attitudes. Secondly, based on the results of the empirical analysis, this paper further discusses the heterogeneity of the impacts of Internet use. The residents with different purposes for using the Internet and different personal characteristics will be affected differently. This study provides a better understanding of the impact of Internet use on residents’ ideologies.

## Data and Methods

### Data

The microdata we use in this paper are from the 2014, 2016, and 2018 CFPS (China Family Panel Studies) database. CFPS is administered by the Institute of Social Science Survey in Peking University. This database survey sample covers 25 provinces, autonomous regions, and municipalities (except Hong Kong, Macao, Taiwan, Xinjiang, Tibet, Qinghai, Inner Mongolia, Ningxia, and Hainan) that represent 95% of the Chinese population. The database includes the social and economic information of individuals and families, and has detailed data of family economic activities, social contacts, demographic statistics, etc. Based on the concerns of this paper, through the consolidation of relevant data, 32,584 data samples were obtained.

### Variables

This paper sets residents’ risk attitudes as the dependent variable. According to the existing research, residents’ risk attitudes can be classified into subjective risk attitudes and objective risk attitudes (Hanna and Chen, 1997). Considering the robustness of the empirical analysis, we also use two methods to measure residents’ risk attitudes. Firstly, based on the response options data in the risk test questionnaire of “Part Q. Behavior and Mental Status” in CFPS 2018, we construct an ordered variable for subjective risk attitudes. According to the degree of risk preference shown in the responses of the surveyed residents, the subjective risk attitudes are measured on a 6-point scale. As the value increases, the degree of risk preference increases. Secondly, we use whether the sample residents hold risky assets to represent their objective risk attitudes. The risky assets here include stocks, bonds, funds, etc. Objective risk attitudes are defined as a dummy variable that equals 1 if the residents hold risky assets, 0 otherwise.

The independent variable is Internet use. Since the purpose of this paper is to analyze the impacts of Internet use on residents’ risk attitudes regarding the Internet as a new information acquisition technology, the measurement of Internet use is based on the answers to the following question: “How importance is Internet as your access to information?” The response options in the questionnaire range from “Very not important = 1” to “Very important = 5.” The values from 1 to 5 represent the increasing importance of the Internet as an access to information. In addition, in the section of robustness analysis, this paper also uses the answers to questions such as “Whether do you use mobile Internet?” and “Whether do you use mobile phone?” to construct alternative variables of Internet use for robustness analysis.

In real life, different people have different purposes for using the Internet. It is thus very natural that we ask the following question: will the different uses of the Internet have different impacts on residents’ risk attitudes? If Internet use can affect the risk attitudes by innovating their access to information, then a reasonable expectation is that different ways of using the Internet will have different impacts on the risk attitudes. Bargh and McKenna (2004) have found that the impact of the Internet on users depends on how they use the Internet. This paper measures the reasons for Internet use based on the following questions: “How important to you in terms of study/work/socializing/entertainment/commercial related activities while using the Internet?” on a scale from “1 = very unimportant, 5 = very important.” This variable is measured on a 5-point scale.

In order to control the influence of other information sources, this paper also uses the answers to the other related questions to construct three traditional information source variables. These related questions in the questionnaire are “How importance is TV/Newspaper and Magazines/From other people as your access to information?” The response options in the questionnaire range from “Very not important = 1” to “Very important = 5.” The values from 1 to 5 represent the increasing importance of these information sources.

Referring to the existing studies, this paper also controls other factors that affect residents’ risk attitudes, such as individual characteristics, including the following: age, gender, health status, education, marital status, study habits, social capital, etc., and other family characteristic variables such as household size, annual income per capita, property value, family debt, etc.

Table 1 reports the statistics of the main variables involved in this paper. On average, residents’ evaluation of the importance of information sources in order from large to small are: TV (the mean value of the importance score is 3.356), The Internet (3.025), Newspapers and magazines (2.584), and Other people’s messages (2.524). Among them, TV, Newspapers and magazines, and Other people’s messages are traditional information sources. This is consistent with our perception in daily life. TV is one of the most popular household appliances at present, and the Internet also plays an increasingly important role in obtaining information. These two points make TV and the Internet become the most two important information sources for residents to obtain information.

**TABLE 1:** Definition and descriptive statistics.

Variables	Definition	Observation	Mean	*SD*
Subjective risk attitudes	Risk preference extent (1–6)	31,304	2.654	1.356
Objective risk attitudes	1 if hold risk assets, 0 otherwise	32,584	0.056	0.167
Internet use	Importance of Internet as an access to information (1–5)	32,011	3.025	1.067
TV	Importance of TV as an access to information (1–5)	32,067	3.356	1.708
Newspapers and magazines	Importance of newspapers and magazines as an access to information (1–5)	32,584	2.584	1.112
Others	Importance of other people’s messages as an access to information (1-5)	32,102	2.524	1.282
Gender	1 = Male, 0 = Female	32,584	0.498	0.500
Age	Age value	32,584	46.250	15.581
Health status	Health Degree (1–3)	32,011	2.125	1.002
Marriage status	1 = Unmarried, 2 = In marriage, 3 = Divorced or widowed	32,584	2.054	0.855
Education	The schooling years	31,005	7.257	5.265
nationality	1 if Han nationality, 0 otherwise	32,584	0.932	0.311
Study habits	1 if has reading habits, 0 otherwise	32,584	0.302	0.410
Language	1 if speaks Mandarin, 0 otherwise	32,584	0.585	0.308
Social relations	Ratio of human consumption to total income	31,065	0.098	0.307
Region	1 = urban, 0 = rural.	30,645	0.499	0.500
Household size	Number of family members	31,765	4.331	3.012
Annual income per capita	Logarithm of annual income per capita	30,653	10.974	1.844
Property	Logarithm of market value of owned property	30,567	13.502	1.553
Liquid assets	Logarithm of total assets (excluding housing) such as cash, bank deposits and stocks	19,087	10.178	1.986
Debt	1 if has family debt, 0 otherwise	32,584	0.157	0.437

*CFPS,China Family Panel Studies.*

### Estimating Methods

There are two empirical models: the subjective risk attitudes impact model and the objective risk attitudes impact model.

The subjective risk attitudes variable is an ordinal variable and have six different levels (1-6). We adopt a Generalized Ordered Logit Model^2^ to discuss how Internet use affects the residents’ subjective risk attitude. The model can be expressed as follows:


(1) $$P\left(R_{i}>j\right)=g\left(X\beta_{j}\right)=\frac{\text{exp}\,\left(\alpha_{j}+X_{i}\,\beta_{j}\right)}{1+\text{exp}\,\left(\alpha_{j}+X_{i}\,\beta_{j}\right)},j=1,2,\ldots ,J-1$$

The probability expression of resident i’s risk attitude is:


$$Prob\left(R_{i}=j\right)=1-g\left(X\beta_{1}\right),j=1$$

$$Prob\left(R_{i}=j\right)=g\left(X\beta_{j-1}\right)-g\left(X\beta_{j}\right),j=2,\ldots ,J-1$$

$$Prob\left(R_{i}=j\right)=g\left(X\beta_{j-1}\right),j=J$$

*Prob*(*R*_*i*_=*j*) is the probability that residents’ risk attitudes variable value is *j*. *J* represents the number of categories for the ordinal risk attitudes variable, here, *J* = 6. When *j* = 1, category 1 is compared with categories 2, 3, 4, and 5; when *j* = 2, category 2 is compared with categories 1, 3, 4, and 5; the interpretations of remaining values are similar. *X* is the set of independent variables including Internet use variable or the reason variables of Internet use.β_*j*_ is the coefficients set of *X*. α is the constant term.

The objective risk attitudes variable is a binary discrete variable. We use a Logit Model to discuss the impact of Internet use on the objective risk attitudes of residents. In this model, the value of objective risk attitudes variable is 0 or 1. And there is a continuous latent variable behind it. This latent variable can be understood as the net utility of risk investment behavior to individuals. When the net utility is positive, individuals choose to hold risk assets; otherwise, individuals will not to hold risk assets. Whether the risk assets are held constitutes the observable value of this potential variable. In this paper, the expressions of latent variable and Logit model are as follows:


(2) $$I\,n\,v\,e\,s\,t_{i}^{*}=\beta_{0}+\beta_{1}\,I\,T\,C_{i}+\beta_{2}\,X_{i}+\varepsilon_{i}$$

(3) $$Prob\left(Invest_{i}=1\right)=Prob\left(Invest_{i}^{*}>0\right)=\Phi \,\left(\beta_{0}+\beta_{1}\,I\,T\,C_{i}+\beta_{2}\,X_{i}+\varepsilon_{i}\right)$$

Where *ITC*_*i*_ represents the Internet use or its reason variables of individual *i*, and *X_i_* is a series of control variables of individual *i* and his or her family. ε_*i*_ is a random disturbance term. The coefficient β_1_ measures the impact of Internet use on the holding behavior of risk assets.

### Addressing the Endogeneity Issue

Our models may have endogeneity problems caused by reverse causation or omitted variables. Firstly, in order to solve the endogeneity problem caused by the possible reverse causality between the core independent variables and the dependent variable, the core independent variables in our models are lagged in time. Specifically, we chose to use the CFPS survey data in 2016 to construct the Internet use variable, the reason variables of Internet use and the traditional information source variables while we use the CFPS survey data in 2018 to construct residents’ risk attitudes variables and other dependent variables. The reason for doing this is as follows. From the second half of year 2014, China’s 4G^3^ technology began to be commercially available on large scale, which popularized various mobile Internet devices. The arrival of the mobile Internet era makes the information technology, represented by the Internet, begin to exert an overall impact on the work and daily lives of all groups in society. The survey data of the CFPS in 2016 can basically reflect the initial states of residents’ use of the Internet as a new source to acquire information after the arrival of the mobile Internet era. That is to say, the Internet use variable is mainly affected by the improvement of the information infrastructure and the popularization of intelligent equipment, but it is not determined by the dependent variable.

However, endogeneity also can stem from the likelihood that some variables may affect Internet use and risk attitudes simultaneously are omitted. In order to further eliminate the questions regarding the endogeneity of the model, following the research of Nie et al. (2017), this paper adopts a two stage least squares (2SLS) approach by using one instrument that is related to Internet use but exogenous to residents’ risk attitudes. We choose the ratio of Internet broadband access terminals^4^ at the provincial or municipality level as the instrumental variable. We employ a probit model with Internet use as a binary variable for the first stage regression and an ordered probit model for the second stage. We use the conditional mixed process (CMP) method proposed by Roodman (2011) to estimate the two-stage model. The first stage regression is:


(4) $$I\,U_{i}=\alpha_{0}+\alpha_{1}\,Z+\alpha_{2}\,X_{i}+\mu_{i}$$

Where *IU*_*i*_ denotes the Internet use variable of individual *i*. *Z* is the instrumental variable. *X_i_* is a vector of the control variables including the traditional information source variables. μ_*i*_ is the error term.

The second stage of this model is:


(5) $$R_{i}=\beta_{0}+\beta_{1}\,\hat{I\,U_{i}}+\beta_{2}\,X_{i}+\varepsilon_{i}$$

Where *R_i_* denotes individual i⁢s′ risk attitude (subjective risk attitude and objective risk attitude). $\hat{I\,U_{i}}$ is the predicted Internet use variable of individual *i* from the first stage regression. *X_i_* is a vector of control variables. ε_*i*_ is the error term.

## Empirical Results

Table 2 shows the estimation results of the General Ordered Logit model with subjective risk attitudes as the dependent variables and the Logit models with the objective risk attitudes as the dependent variables. To provide a visual representation of the specific effect of a one-unit change in the independent variable on the dependent variable, the table lists the odds ratios.

**TABLE 2:** Estimates of the impact of internet use on residents’ risk attitudes.

Variables	Odds ratio						
	
	Subjective risk attitudes model					Objective risk attitudes model	
		
	(1)	(2)	(3)	(4)	(5)	(7)	(8)
	Group 1	Group 2	Group 3	Group 4	Group 5		
Internet use	1.1125***	1.0614***	1.0709***	1.0728***	1.0598**	1.2211***	1.2361***
	(0.0180)	(0.0186)	(0.0212)	(0.0240)	(0.0270)	(0.0435)	(0.0449)
TV	0.9508***	0.9847	0.9818	0.9837	0.9841		0.9257**
	(0.0154)	(0.0171)	(0.0192)	(0.0218)	(0.0250)		(0.0336)
Newspapers and magazines	1.0092	1.0390*	1.0470**	1.0445*	1.0739**		0.9523
	(0.0193)	(0.0211)	(0.0238)	(0.0266)	(0.0312)		(0.0375)
Others	0.9994	0.9994	0.9994	0.9994	0.9994		0.9735
	(0.0151)	(0.0151)	(0.0151)	(0.0151)	(0.0151)		(0.0352)
Gender	1.2094***	1.2709***	1.4404***	1.5795***	1.4052***	0.8419**	0.8385**
	(0.0492)	(0.0553)	(0.0711)	(0.0873)	(0.0899)	(0.0729)	(0.0729)
Age	0.9741***	0.9750***	0.9747***	0.9693***	0.9753***	1.0204***	1.0244***
	(0.0019)	(0.0020)	(0.0022)	(0.0026)	(0.0030)	(0.0040)	(0.0043)
**Health status**							
Average	1.0244***	0.9647***	0.9751***	0.9745***	0.9693***	1.0742***	1.0320*
	(0.0043)	(0.0019)	(0.0020)	(0.0022)	(0.0026)	(0.0030)	(0.0152)
Healthy	1.0845***	1.0820	1.1866**	1.0620***	1.0537**	1.0809**	1.0728**
	(0.0141)	(0.0055)	(0.0058)	(0.0065)	(0.0073)	(0.0085)	(0.0111)
**Marriage status**							
In marriage	1.0175*	1.0175*	1.0175*	1.0175*	1.0175*	0.9854	0.9873
	(0.0090)	(0.0090)	(0.0090)	(0.0090)	(0.0090)	(0.0196)	(0.0198)
Divorced or widowed	0.9501	0.9052**	0.9916	1.0188	0.9807	0.9866	0.9782
	(0.0377)	(0.0367)	(0.0441)	(0.0493)	(0.0550)	(0.0780)	(0.0775)
Education	1.0020	1.0866**	1.0820***	1.0837**	1.0809**	1.0843***	1.0845***
	(0.0055)	(0.0058)	(0.0065)	(0.0073)	(0.0085)	(0.0139)	(0.0141)
Nationality	0.9312	0.9312	0.9312	0.9312	0.9312	1.2046	1.1986
	(0.1598)	(0.1598)	(0.1598)	(0.1598)	(0.1598)	(0.5986)	(0.5943)
Study habits	1.1373**	1.0986*	0.9443*	1.0402	0.9197*	1.5064***	1.5167***
	(0.0598)	(0.0612)	(0.1128)	(0.0715)	(0.1414)	(0.1436)	(0.1469)
Region	0.9468***	0.9750***	0.9660***	0.9847***	0.9894***	0.9763***	0.9851*
	(0.0049)	(0.0017)	(0.0020)	(0.0026)	(0.0021)	(0.0029)	(0.0086)
Social relations	1.3707***	1.3707***	1.3707***	1.3707***	1.3707***	0.1384***	0.1499***
	(0.1141)	(0.1141)	(0.1141)	(0.1141)	(0.1141)	(0.0989)	(0.1062)
Household size	1.0105	1.0156	0.9910	0.9846	0.9722	0.9639	0.9627
	(0.0114)	(0.0121)	(0.0133)	(0.0150)	(0.0169)	(0.0255)	(0.0256)
Income per capita	1.0789***	1.0789***	1.0789***	1.0789***	1.0789***	1.8388***	1.8230***
	(0.0293)	(0.0293)	(0.0293)	(0.0293)	(0.0293)	(0.1229)	(0.1218)
Property	1.0133	0.9923	1.0181	1.0291	1.0186	1.7714***	1.7696***
	(0.0172)	(0.0180)	(0.0208)	(0.0237)	(0.0268)	(0.0695)	(0.0696)
Liquid assets	1.0282**	1.0124	0.9920	0.9950	0.9809	1.3295***	1.3316***
	(0.0129)	(0.0135)	(0.0148)	(0.0167)	(0.0184)	(0.0422)	(0.0424)
Debt	1.1176*	1.1176*	1.1176*	1.1176*	1.1176*	0.7960	0.7792
	(0.0710)	(0.0710)	(0.0710)	(0.0710)	(0.0710)	(0.1690)	(0.1659)
Constant	0.4992**	0.5056**	0.2613***	0.1747***	0.1652***	0.0000***	0.0000***
	(0.1386)	(0.1464)	(0.0817)	(0.0597)	(0.0626)	(0.0000)	(0.0000)
Pseudo *R*^2^			0.0472			0.3210	0.3226
Prob > Chi^2^			0.0000			0.0000	0.0000
Observations	25,762	25,762	25,762	25,762	25,762	24,245	24,245

The coefficients reported in this table are **odds ratios** of all variables; standard errors are in parentheses. ***, **, * indicate significance at the 1, 5, and 10% level, respectively.

### Impacts of Internet Use on Residents’ Subjective Risk Attitudes

The regression results show that the subjective risk attitude variable is significantly positively correlated with the Internet use variable in every risk attitudes group. In columns 1–5, the odds ratios are significance at the 1 or 5% level. This suggests that Internet use, which is a technological innovation of information acquisition source can indeed improve the residents’ subjective risk preferences.

As for the three traditional information sources, there is no significant correlation between “TV” and residents’ subjective risk attitudes. The odds ratios of “Newspapers and magazines” in most groups are significantly positive at the 5 or 10% significance level while the odds ratios of “other people’s messages” in all groups are not significant. This implies that: (1) TV, as a source for residents to obtain information, cannot affect their subjective risk attitudes by improving their ability to obtain information. There are two possible reasons. TV programs are usually broadcasted regularly, and so residents obtain information passively and cannot actively choose the information they need. This leads to the result that the residents’ sense of information acquisition is not strong. Also, in daily life, watching TV is usually regarded as a kind of leisure activity. TV has little impact on improving the ability of residents to obtain useful information. Therefore, TV plays a very limited role in influencing the residents’ subjective risk attitudes. (2) Newspapers and magazines usually contain a lot of formal information about politics, the economy, society, and production. Residents can actively choose the information they need. Therefore, this information source improves the ability of residents to acquire “formal knowledge,” which helps to improve their awareness of themselves and their situation. In other words, the dependence on this information source improves their risk preference. (3) If residents rely on too much “other people’s messages” to get information, it is not conducive to alleviating their risk aversion attitudes. In daily life, we sometimes have the experience that when we share our “new ideas” and “new plans” with others, we usually experience “poured by cold water” instead of being encouraged and supported. Therefore, relying too much on others’ messages to obtain information is not conducive to improving their subjective risk preferences.

Regarding the control variables, Gender, Health status, In marriage, Study habits, Social relations, Annual income per capita, and Family debt, etc. all have significant positive correlations with the subjective risk attitudes variables in various significant degrees.

### Impacts of Internet Use on Residents’ Objective Risk Attitudes

Referring to the methods of relative literatures, this paper uses whether residents hold risky assets to represent the residents’ objective risk attitudes. The risky assets mentioned in this paper are financial assets including stocks, funds, treasury bonds, trust products, and foreign exchange products. We use the Logit Model to analyze the relationship between Internet use and residents’ objective risk attitudes. According to the regression results in columns (7) and (8) of Table 2, regardless of whether the traditional information source variables are controlled or not, the objective risk attitudes variables are significantly positively correlated with the Internet use variable at 1% significance level.

This shows that the innovation of information sources has also improved the objective risk preferences of residents. The higher the dependence on the Internet to obtain information is, the higher the objective risk preferences are. In addition, the coefficients of the three traditional information source variables are not significant, indicating that the impacts of the Internet on risk attitudes is far greater than those of traditional information sources.

Regarding the control variables, Age, Health status, Education, Study habits, Annual income per capita, The value of family Property, and Family Liquid assets, etc. all have significant positive correlations with the objective risk attitudes variable. However, the coefficients of Gender and Social relations are significantly negative.^5^

## Heterogeneity Analysis

Up to this point, we have verified the positive relationship between internet use and residents’ risk attitudes. In this paper, the Internet use variable is expressed in terms of the importance evaluation of the Internet as an access to information. We don’t need to do the mechanism analysis like other studies, because the connotation of Internet use variable has precisely pointed out this logic: The Internet has innovated our mode of acquiring information. In the process of obtaining information, the more we rely on the Internet, the greater its impact on our concept is.

Next, we are interested in whether the positive impacts of Internet use are different among groups with different characteristics. In previous part of this paper, we take the generalized ordered logit model as our analytical tool to discuss the relationship between Internet use and residents’ subjective risk attitudes. As the subjective risk attitudes variables are divided into 6 levels, this leads to the parallel line hypothesis required by ordered logit model is not satisfied. In order to facilitate the following subgroup regression analysis, we reorganize the subjective risk attitudes variable, and measure it on a 2-point scale: 0 = Low risk preference, 1 = High risk preference.^6^ Then we can estimate the subjective risk attitudes model with a logit model by using the subgroup data to analysis the heterogeneous impacts of Internet use. As before, the following regression results also report the odds ratios.

### Internet Use Reasons and Risk Attitudes

The reasons for Internet use indicate residents’ motivations that can help us better understand the relationship between Internet use and risk attitudes. If Internet use can alleviate the risk aversion of residents by improving their access to information, then a reasonable expectation is that different ways of using the Internet will have different impacts on their risk attitudes. In the following models, we group the whole samples based on their frequency data of using the Internet for study, work, socializing, entertainment, and commercial related activities. Considering the heterogeneity of usage, we empirically analyze the impacts of the Internet on residents’ risk attitudes. Tables 3–7 show the regression results of the different grouping models.

**TABLE 3:** Grouped by the using frequency for studying.

Variable	Subjective risk attitudes		Objective risk attitudes	
		
	High	Low	High	Low
Internet use	1.0874*** (0.0264)	1.0465 (0.0397)	1.3231*** (0.0599)	0.9679 (0.0658)
TV	1.0004 (0.0239)	0.9277** (0.0336)	0.9025** (0.0403)	0.9855 (0.0627)
New. and mag.	1.0518* (0.0289)	1.0443 (0.0452)	0.9695 (0.0463)	0.9268 (0.0674)
Others	0.9665 (0.0222)	1.0028 (0.0367)	0.9796 (0.0430)	0.9603 (0.0635)
Individual characteristics	Yes	Yes	Yes	Yes
Family characteristic	Yes	Yes	Yes	Yes
Observations	10,345	4,578	11,234	5,460
Pseudo *R*^2^	0.0331	0.0246	0.3407	0.3032

The coefficients reported in this table are odds ratios of all variables; standard errors are in parentheses. ***, **, * indicate significance at the 1, 5, and 10% level, respectively.

**TABLE 4:** Grouped by the using frequency for work.

Variable	Subjective risk attitudes		Objective risk attitudes	
		
	High	Low	High	Low
Internet use	1.0922*** (0.0247)	1.0412 (0.0451)	1.3619*** (0.0495)	1.1389 (0.1145)
TV	0.9795 (0.0221)	0.9655 (0.0408)	0.9435 (0.0374)	0.8614 (0.0805)
New. and mag.	1.0501* (0.0272)	1.0482 (0.0526)	0.9701 (0.0408)	0.8174* (0.0971)
Others	0.9826 (0.0215)	0.9510 (0.0402)	0.9809 (0.0385)	0.9123 (0.0862)
Individual characteristics	Yes	Yes	Yes	Yes
Family characteristic	Yes	Yes	Yes	Yes
Observations	11,306	3,578	9,834	3,860
Pseudo *R*^2^	0.0332	0.0192	0.3342	0.3232

The coefficients reported in this table are odds ratios of all variables; standard errors are in parentheses. ***, * indicate significance at the 1, 5, and 10% level, respectively.

**TABLE 5:** Grouped by the using frequency for socializing.

Variable	Subjective risk attitudes		Objective risk attitudes	
		
	High	Low	High	Low
Internet use	1.0698*** (0.0239)	1.0646 (0.0478)	1.3046*** (0.0550)	0.9518 (0.0748)
TV	0.9701 (0.0216)	1.0063 (0.0436)	0.9336* (0.0392)	0.9049 (0.0673)
New. and mag.	1.0486* (0.0274)	1.0543 (0.0516)	0.9421 (0.0427)	0.9842 (0.0818)
Others	0.9852 (0.0213)	0.9496 (0.0421)	0.9779 (0.0405)	0.9907 (0.0777)
Individual characteristics	Yes	Yes	Yes	Yes
Family characteristic	Yes	Yes	Yes	Yes
Observations	15,125	1,655	15,262	1,658
Pseudo *R*^2^	0.0396	0.0162	0.3280	0.3241

The coefficients reported in this table are odds ratios of all variables; standard errors are in parentheses. ***, * indicate significance at the 1, 5, and 10% level, respectively.

**TABLE 6:** Grouped by the using frequency for entertainment.

Variable	Subjective risk attitudes		Objective risk attitudes	
		
	High	Low	High	Low
Internet use	1.0764*** (0.0233)	1.0699 (0.0526)	1.3005*** (0.0544)	0.9758 (0.0764)
TV	0.9657 (0.0213)	1.0181 (0.0465)	0.9495 (0.0403)	0.8774* (0.0627)
New. and mag.	1.0603** (0.0270)	1.0169 (0.0543)	0.9710 (0.0438)	0.8747 (0.0726)
Others	0.9795 (0.0208)	0.9559 (0.0461)	0.9432 (0.0391)	1.0519* (0.0805)
Individual characteristics	Yes	Yes	Yes	Yes
Family characteristic	Yes	Yes	Yes	Yes
Observations	14,089	1,851	15,063	1,857
Pseudo *R*^2^	0.0391	0.0192	0.3403	0.2682

The coefficients reported in this table are odds ratios of all variables; standard errors are in parentheses. ***, **, * indicate significance at the 1, 5, and 10% level, respectively.

**TABLE 7:** Grouped by the using frequency for commerce.

Variable	Subjective risk attitudes		Objective risk attitudes	
		
	High	Low	High	Low
Internet use	1.0941*** (0.0260)	1.0606 (0.0424)	1.3146*** (0.0592)	1.0026 (0.0708)
TV	0.9819 (0.0229)	0.9613 (0.0367)	0.9151 (0.0401)	0.9725 (0.0649)
New. and mag.	1.0441 (0.0283)	1.0628 (0.0463)	0.9958 (0.0468)	0.8476 (0.0622)
Others	0.9916 (0.0223)	0.9303* (0.0360)	0.9876 (0.0423)	0.9566 (0.0661)
Individual characteristics	Yes	Yes	Yes	Yes
Family characteristic	Yes	Yes	Yes	Yes
Observations	12,486	4,343	12,564	4,356
Pseudo *R*^2^	0.0389	0.0230	0.3382	0.2966

The coefficients reported in this table are odds ratios of all variables; standard errors are in parentheses. ***, * indicate significance at the 1, 5, and 10% level, respectively.

It can be seen from the results of all the models above that although there are different reasons for using the Internet, the higher the frequency of internet use, the more significant the positive impacts of Internet use on their risk attitudes. For the group with low frequency of Internet use, the coefficients of the Internet use variables in most models are not significant or negative. Further comparing the variable coefficients of each model, we also find that the positive impacts of Internet use are greater in those groups who use the Internet for commerce, study, and work activities.

### Internet Use, Personal Characteristics, and Risk Attitudes

According to the previous literature review, individual cognition, learning ability, and life events experience, etc. are all important factors affecting individual risk attitudes. Although we have discussed the impacts of different reasons for Internet use, we have not discussed the impacts of different individual characteristics. Regarding the heterogeneity of individual characteristics, the following part of this paper examines which kind of residents’ risk attitudes are more affected by Internet use. In the following part, the sample residents are divided into groups according to their study motivation, study ability, region, and life events experience. By doing this, we analysis the impacts of Internet use on the risk attitudes of residents with different personal characteristics. Tables 8–11 show the regression results of the different grouping models.

**TABLE 8:** Heterogeneity of the learning initiative.

Variable	Subjective risk attitudes		Objective risk attitudes	
		
	Have reading habits	Have no reading habits	Have reading habits	Have no reading habits
Internet use	1.0831*** (0.0236)	1.0517 (0.0482)	1.2302*** (0.0800)	1.2473*** (0.0553)
TV	0.9751 (0.0219)	0.9858 (0.0417)	0.9027* (0.0524)	0.9502 (0.0448)
New. and mag.	1.0817*** (0.0289)	0.9688 (0.0440)	0.8989* (0.0559)	0.9948 (0.0508)
Others	0.9712 (0.0211)	0.9979 (0.0432)	0.9914 (0.0591)	0.9624 (0.0439)
Individual characteristics	Yes	Yes	Yes	Yes
Family characteristic	Yes	Yes	Yes	Yes
Observations	4,616	13,146	4,616	13,146
Pseudo *R*^2^	0.0313	0.0398	0.2785	0.3012

The coefficients reported in this table are odds ratios of all variables; standard errors are in parentheses. ***, * indicate significance at the 1, 5, and 10% level, respectively.

**TABLE 9:** Heterogeneity of the learning ability.

Variable	Subjective risk attitudes		Objective risk attitudes	
		
	More educated	Less educated	More educated	Less educated
Internet use	1.0758*** (0.0277)	1.0567 (0.0618)	1.3145*** (0.0537)	0.0906 (0.0919)
TV	0.9676 (0.0252)	0.9911 (0.0300)	0.8754*** (0.0349)	1.0431 (0.0897)
New. and mag.	1.0523* (0.0302)	1.0334 (0.0398)	1.0016 (0.0430)	1.1049 (0.0948)
Others	0.9715 (0.0254)	0.9908 (0.0282)	0.9235* (0.0380)	1.0414 (0.0768)
Individual characteristics	Yes	Yes	Yes	Yes
Family characteristic	Yes	Yes	Yes	Yes
Observations	9,168	6,594	9,168	6,594
Pseudo *R*^2^	0.0333	0.0287	0.2687	0.3281

The coefficients reported in this table are odds ratios of all variables; standard errors are in parentheses. ***, * indicate significance at the 1, 5, and 10% level, respectively.

**TABLE 10:** Mean test.

	Have no life events			Have life events			Difference
		
	N	Mean	*SD*	N	Mean	*SD*	
Subjective risk attitudes	17,173	2.5005	1.7771	4,755	2.0407	1.7069	0.4596***
Objective risk attitudes	17,173	0.0674	0.2326	4,755	0.0542	0.2265	0.0138**

***, **, indicate significance at the 1, 5, and 10% level, respectively.

We use the empirical analysis approach to explore the marginal impact of Internet use on the risk attitudes of these two types of residents. Table 11 reports the regression results. The results show that compared with the residents without life events experience, Internet use can more significantly improve the risk attitudes of the residents with life events experience.

**TABLE 11:** Heterogeneity of life experience.

Variable	Subjective risk attitudes		Objective risk attitudes	
		
	Have life events	Have no life events	Have life events	Have no life events
Internet	1.0947** (0.0488)	1.0057 (0.0234)	1.3626*** (0.1012)	1.1969* (0.0500)
TV	1.0319 (0.0457)	0.9630* (0.0213)	0.9584 (0.0729)	0.9188** (0.038 1)
New. and mag.	0.9980 (0.0515)	1.0644** (0.0273)	0.8695 (0.0750)	0.9801 (0.0437)
Others	1.0283 (0.0453)	0.9665 (0.0209)	0.8615* (0.0671)	1.0023 (0.0413)
Individual characteristics	Yes	Yes	Yes	Yes
Family characteristic	Yes	Yes	Yes	Yes
Observations	2,775	13,188	2,812	13,776
Pseudo *R*^2^	0.0404	0.0335	0.3076	0.3315

The coefficients reported in this table are odds ratios of all variables; standard errors are in parentheses. ***, **, * indicate significance at the 1, 5, and 10% level, respectively.

As a new type of information technology, the impact of Internet use requires its users to have some learning initiatives. A reasonable expectation is that the impact of Internet use on the risk attitudes of residents with strong learning initiatives is more significant. Whether having the habit of reading can be used as an evaluation criterion of residents’ learning initiatives. The overall sample is divided into the group with reading habits and the group without reading habits. Then, we empirically analyze the two groups. Table 8 shows the regression results. For residents with active learning habits, the coefficients of the Internet use variable are significantly positive at 1% significance level for both the subjective risk attitudes model and the objective risk attitudes model; and for the residents without active learning habits, the coefficients of the Internet use variable are not significant in subjective risk attitudes model. But in the objective risk attitudes model, the coefficients of both groups are significantly positive. One reason might be related to the measurement method of the objective risk attitudes, the second is that whether having reading habits actually cannot reflect residents’ learning abilities (Precisely, the application of a new technology and the reverse impact of new technology are highly related to the learning ability of users).

Considering the results, we find that for residents without active learning habits, Internet use does not have a significant impact on their subjective risk attitudes. Meanwhile, grouping does not affect the positive influence of Internet use on objective risk attitudes.

Although the results of the above models show that Internet use has different effects on the risk attitudes of residents with different learning initiatives, from a more accurate point of view, the influencing degree depends on the users’ learning and acceptance abilities. Because with the continued prosperity of the Internet Ecology, the information provided by the Internet is increasingly abundant. The influence of information sources on the concept of residents is closely related to their learning and comprehension abilities. Residents with strong learning and comprehension abilities can better tap the intrinsic value of the rich information connected by the Internet, and their thoughts are affected more deeply. Therefore, the regression model shown in Table 8 above actually implies a hypothesis that the residents with active learning habits have higher learning abilities. In order to measure the learning ability more accurately, the following takes the actual years of education as the measurement of their learning abilities. The reasons are as follows: first, the greater the number of years of education is, the better the learning ability; and second, the stronger the learning ability is, the more likely the individual is to receive formal education for a longer time. Therefore, the actual years of education can accurately measure the learning abilities of residents.

In the following model, the overall sample is divided into a group with relatively more education years and a group with relatively fewer education years.^7^ Table 9 shows the regression results of the two groups. Internet use has significantly improved the subjective and objective risk attitudes of the more educated residents, but overall does not have a significant impact on the risk attitudes of the less educated residents.

The studies of Dohmen et al. (2016) and Banks et al. (2019) both found that events in life have significant effects on individual risk preferences. According to whether the residents have been hospitalized or unemployed for more than 12 months in 2018 and the past 5 years, we construct a proxy variable of personal life events experience^8^ by summarizing the survey data of CFPS 2014, CFPS 2016, and CFPS 2018. Based on this dummy variable, the samples were divided into two groups: those with life events experience and those without life events experience. First of all, Table 10 reports the mean test result of the subjective and objective risk attitudes variables of the two groups of residents. It can be seen that the residents with life events experience are more conservative than the residents without life events experience from both subjective and objective aspects. This implies that life events experience tends to make individuals risk averse. This can be due to the fact that after experiencing a life change, people will generate a fear of uncertainty in the future, which makes them more risk averse.

The mean test result in Table 10 has shown that the residents with life events experience are more conservative than those without life events experience. In Table 11, we find that for the residents with life events experience, the marginal impacts of Internet use on their risk attitudes are more significant. These two facts show that Internet use can offset the negative impacts of life events experience on the risk attitudes to a certain extent. These results confirm that Internet use can alleviate residents’ fear of uncertainty and improve their risk preferences.

## Robustness Analysis

### Endogeneity

We report the IV estimates for the subjective and objective risk attitudes in Table 12.^9^ We use the ratio of Internet broadband access users in the total population at the provincial or municipality level as the instrumental variable and the results show that regardless of whether other information sources are controlled or not, the coefficients of the instrumental variable are all significantly positive in both the two kinds of models. This is consistent with the previous findings.

**TABLE 12:** Conditional mixed process (CMP) 2SLS estimates of the effect of Internet use on risk attitudes.

Variable	Subjective risk attitudes model	Objective risk attitudes model
Internet use	0.8226*	0.9954**
	(0.2382)	(0.3175)
Instrumental variable	0.2405*** (0.0495)	0.2659*** (0.0473)
Other information sources	Yes	Yes
Individual characteristics	Yes	Yes
Family characteristics	Yes	Yes
Wald Chi2	237.92	1155.19
Observations	25489	25489

This table reports the original coefficients of the models, standard errors are in parentheses. The dependent variables are subjective risk attitudes (measured on a 6-point scale) and objective risk attitudes (measured on a binary variable). We use the conditional mixed process (CMP) method to estimate the IV-probit models. ***, **, * indicate significance at the 1, 5, and 10% level, respectively.

### Using Selection on Observables to Assess the Bias From Unobservables

Even though we tried to control many individual and family characteristics in our identification strategy, there still may be the concern that some unobservable variables correlated with Internet use and residents’ risk attitudes may bias our results. How can we assess the bias caused by this problem? Following the studies of Nunn and Wantchekon (2011) and Oster (2015), we use a method to examine to what extent the unobservable variables may bias the results above. Specifically, this method constructs a ratio value through the results of different forms of regression models constructed by observable variables. This ratio value reflects how important the unobservable variables need to be to bias the original results. Through this method, we find that the unobservable variables have to be 6.3 times as important as the observable variables to have a significant impact on the original results. From this perspective, the possibility of a significant deviation caused by unobservable variables is very small. It is unlikely that the possible unobservable factors are a fatal problem.

### Adjusting the Variables, Samples, and Models

In order to verify the impacts of Internet use from multiple perspectives, this paper also uses the data from the CFPS questionnaires to construct alternative variables of information technology applications. We use these alternative variables to replace the Internet use variable in the previous empirical models for a robustness test. The empirical results show that the substitute information technology variables still have significant positive effects on residents’ risk attitudes.

Further, we also process the model data, and then put the processed data into the original regression models to test the robustness of the results. Specifically, first of all, considering that there are differences in the concepts of the elderly and young adults, in the processed data, we exclude the samples of residents aged 60 and above. Secondly, in order to avoid the effects of outliers in the sample data on the regression results of the model, we also winsorize the continuous variables at the 1 and 99% levels. In order to discuss the non-linear relationship between age and risk attitude, we also add the squared variable of age to the model. Finally, we put the above data into the regression models, and find that the positive impact of Internet use on residents’ risk attitudes has not changed.

## Conclusion and Discussion

### Conclusion

Nowadays, the popularization and application of information technology in China plays an increasingly important role in promoting economic and social development. In essence, the innovation of information technology represented by the Internet has changed the mode of residents’ information access. This change has had a great impact on residents’ concepts. From the perspective of residents’ risk attitudes, this paper empirically analyzes the impacts of Internet use on residents’ risk attitudes. The results show the following. Firstly, Internet use can significantly improve residents’ subjective and objective risk preference attitudes. Secondly, this paper further analyzes the impact of Internet use on risk attitudes with different reasons for use. The results show that although there are different purposes for using the Internet, the common thing is that in the process of obtaining information, the higher the dependence on Internet use is, the more significant the positive impact of Internet use on their risk attitudes. Finally, this paper also considers the heterogeneity of residents’ personal characteristics and finds that Internet use is more helpful to enhance the risk preference attitudes of those residents who have active learning habits, more years of education, more wealth, and more life events experience. This study provides systematic evidence for the relationship between Internet use and residents’ risk attitudes. Risk attitudes are an important factor affecting residents’ savings, consumption, investment, labor supply, insurance and health services purchases and many other economic behaviors. The changes of residents’ risk attitudes are conducive to improving the tolerance of micro subjects to the overall economic risk and encouraging innovation activities in society, which is greatly conductive to high-quality development.

### Policy Implications

The Chinese government has launched its strategy “Digital China” to facilitate the information of the whole society. This strategy aims to improve the economic efficiency and social innovation through the construction of information infrastructure and the application of information technology. As one of the most important factors affecting residents’ behaviors, risk attitudes not only play a role at the micro level, but also are closely linked with the innovation ability of the whole society at the macro level. Improving the risk preference of the whole society is conductive to stimulating the innovation vitality of our society. Given the significant positive impact of Internet use on residents’ risk attitudes, the government should promote the construction of the information infrastructure and implement a digital development strategy. While promoting the application and popularization of information technology, we should pay attention to guiding the residents’ habits when using information technology so as to give full play to the positive impacts of information technology. We should also pay attention to the individual characteristics of residents, strengthen the cultural construction, and promote the growth of residents’ income and wealth so that residents can better share the “digital dividend” brought by the popularization of information technology.

## Data Availability Statement

Publicly available datasets were analyzed in this study. This data can be found here: http://www.isss.pku.edu.cn/cfps/download.

## Author Contributions

SZ, GZ, and JL: conceptualization, data collection, analysis, and manuscript preparation. SZ, GZ, and HG: conceptualization and manuscript preparation. All authors contributed to the article and approved the submitted version.

## Conflict of Interest

The authors declare that the research was conducted in the absence of any commercial or financial relationships that could be construed as a potential conflict of interest.

## Publisher’s Note

All claims expressed in this article are solely those of the authors and do not necessarily represent those of their affiliated organizations, or those of the publisher, the editors and the reviewers. Any product that may be evaluated in this article, or claim that may be made by its manufacturer, is not guaranteed or endorsed by the publisher.

## Footnotes

1 Data source: China Internet Network Information Center, 2020.

2 The Ordered Logit/Probit Model needs the dependent variable satisfy the parallel line hypothesis which is difficult for real-world data. So we use Generalized Ordered Logit Model which relaxes the condition and can redress the shortcomings of Ordered Logit/Probit Model.

3 “4G” is “The 4th generation mobile communication technology.” It has a faster Internet access speed and brings the era of mobile Internet.

4 The data are from “China Statistical Yearbook 2016.”

5 Table 2 reports the odds ratios of all variables. The value of odds ratio < 1 means that the independent variable is negatively correlated with the dependent variable.

6 If the risk attitudes value is smaller than the mean value, we group the samples as Low risk preference. If the risk attitudes value is greater than the mean value, we group the samples as High risk preference.

7 If the schooling year value is greater than the mean value, we group the samples as More educated. If the schooling years is greater than the mean value, we group the samples as Less educated.

8 If we consider the experience of hospitalization or unemployment, respectively, the sample size of both is small. Furthermore, the two kinds of experiences are both setbacks in life. Here, we combine these two kinds of life experiences to construct the personal experience variable.

9 We employ a IV-probit model to do the endogenous analysis.

## References

[B1] Arrondel L. Lefebvre B. (2001). Behavior of household portfolios in france: the role of housing. *Rev. Income Wealth* 47 489–514. 10.1111/1475-4991.00031

[B2] Banks J. Bassoli E. Mammi I. (2019). Changing attitudes to risk at older ages: the role of health and other life events. *J. Econ. Psychol.* 79 1–20. 10.1016/j.joep.2019.102208

[B3] Bargh J. A. McKenna K. Y. A. (2004). “The internet and social life,”. *Ann. Rev. Psychol.* 55 573–590. 10.1146/annurev.psych.55.090902.141922 14744227

[B4] Bonsang E. Dohmen T. (2015). Risk attitude and cognitive aging. *J. Econ. Behav. Organiz.* 112 112–126. 10.1016/j.jebo.2015.01.004

[B5] Bucciol A. Zarri L. (2017). Do personality traits influence investors’ portfolios? *J. Behav. Exp. Econ.* 68 1–12. 10.1016/j.socec.2017.03.001

[B6] Camerer C. F. Hogarth R. M. (1999). The effects of financial incentives in experiments: a review and capital-labor-production framework. *J. Risk Uncertaint.* 19 7–42. 10.1023/A:1007850605129

[B7] Chen M. K. (2013). The effect of language on economic behavior: evidence from savings rates, health behaviors, and retirement assets. *Am. Econ. Rev.* 103 690–731. 10.1257/aer.103.2.690 29524925

[B8] Cho I. Orazem P. F. Rosenblat T. (2018). Are risk attitudes fixed factors or fleeting feelings. *J. Labor Res.* 39 127–149. 10.1007/s12122-018-9262-2

[B9] Cocco J. F. (2005). Portfolio choice in the presence of housing. *Rev. Finan. Stud.* 18 535–567. 10.1093/rfs/hhi006

[B10] Dohmen J. T. Falk A. Huffman D. Sunde U. Schupp J. Wagner G. G. (2011). Individual risk attitudes: measurement, determinants, and behavioral consequences. *J. Eur. Econ. Assoc.* 9 522–550. 10.1111/j.1542-4774.2011.01015.x

[B11] Dohmen T. Lehmann H. Pignatti N. (2016). Time-varying individual risk attitudes over the Great Recession: a comparison of Germany and Ukraine. *J. Compar. Econ.* 44 182–200. 10.1016/j.jce.2015.10.002

[B12] Fenghua L. (2013). ““Students risk attitudes in college choice game under information constraint,”,” in *International Conference on Information, Business and Education Technology*, (Amsterdam: Atlantis Press).

[B13] Gary C. Angelino V. (2012). Three risk-elicitation methods in the field: evidence from rural Senegal. *Rev. Behav. Econ.* 3 145–171. 10.1561/105.00000046

[B14] Ghadim A. K. Pannell D. J. Burton M. P. (2005). Risk, uncertainty, and learning in adoption of a crop innovation. *Agricult. Econ.* 33 1–9. 10.1111/j.1574-0862.2005.00433.x

[B15] Gollier C. (2002). Time diversification, liquidity constraints, and decreasing aversion to risk on wealth. *J. Monetar. Econ.* 49 1439–1459. 10.1016/S0304-3932(02)00173-3

[B16] Guiso L. Paiella M. (2008). “Risk aversion, wealth, and background risk,”. *J. Eur. Econ. Assoc.* 6 1109–1150. 10.1162/JEEA.2008.6.6.1109

[B17] Haliassos M. Bertaut C. C. (1995). “Why do so few hold stocks?”. *Econ. J.* 105 110–1129. 10.14283/jpad.2016.118 29199321

[B18] Hammitt J. Haninger K. Treich N. (2009). Effects of health and longevity on financial risk tolerance. *Genev. Risk Insurance Rev.* 34 117–139. 10.1057/grir.2009.6

[B19] Hanna S. D. Chen P. (1997). Subjective And Objective Risk Tolerance: implications For Optimal Portfolios. *Finan. Counsel. Planning* 2 17–26. 10.2139/ssrn.95488

[B20] Honarvar B. Lankarani K. B. Kharmandar A. (2020). “Knowledge, attitudes, risk perceptions, and practices of adults toward COVID-19: a population and field-based study from Iran. *Int. J. Public Health* 65 731–739. 10.1007/s00038-020-01406-2 32583009 PMC7311321

[B21] Jung S. Treibich C. (2015). “Is self-reported risk aversion time variant?”. *Revue D Economie Politique* 125 547–570. 10.1001/archgenpsychiatry.2008.3 18678793 PMC3782742

[B22] Kapteyn A. Teppa F. (2011). Subjective measures of risk aversion, fixed costs, and portfolio choice. *J. Econ. Psychol.* 32 564–580. 10.1016/j.joep.2011.04.002

[B23] Nie P. Sousa-Poza A. Nimrod G. (2017). Internet use and subjective well-being in China. *Soc. Indic. Res.* 132 489–516. 10.1007/s11205-015-1227-8

[B24] Nunn N. Wantchekon L. (2011). “The slave trade and the origins of mistrust in Africa.”. *Am. Econ. Rev.* 101 3221–3252. 10.1257/aer.101.7.3221

[B25] Oster E. (2015). “Unobservable selection and coefficient stability: Theory and validation,”,” in *NBER Working Paper No. w19054.* Available online at SSRN: https://ssrn.com/abstract=2266720 (accessed July 21, 2019).

[B26] Outreville J. F. (2013). The relationship between relative risk aversion and the level of education: a survey and implications for the demand for life insurance. *J. Econ. Surveys* 29 97–111. 10.1111/joes.12050

[B27] Sahm C. R. (2012). How much does risk tolerance change. *Quarter. J. Finan.* 2 1–38. 10.1142/S2010139212500206 25544881 PMC4276321

[B28] Tausch F. Zumbuehl M. (2018). “Stability of risk attitudes and media coverage of economic news,”. *J. Econ. Behav. Organ.* 150 295–310. 10.1016/j.jebo.2018.01.013

[B29] Wang Q. Li M. (2012). Home computer ownership and Internet use in China: trends, disparities, socioeconomic impacts, and policy implications,”. *First Monday* 17 1–15. 10.5210/fm.v17i2.3767

[B30] Wijayaratna K. P. Dixit V. (2016). Impact of information on risk attitudes: implications on valuation of reliability and information. *J. Choice Model.* 20 16–34. 10.1016/j.jocm.2016.09.004

[B31] Wossen T. Berger T. Falco D. S. (2015). Social capital, risk preference and adoption of improved farm land management practices in Ethiopia. *Agricult. Econ.* 46 81–97. 10.1111/agec.12142

